# Supplementary material for: Barriers for health care access affects maternal continuum of care utilization in Ethiopia; spatial analysis and generalized estimating equation
Source: PLoS One. 2022 Apr 22;17(4):e0266490. doi: 10.1371/journal.pone.0266490 (PMC9032438; doi:10.1371/journal.pone.0266490)
Supplement: Attachment — Submitted filename: Response to Reviewers.docx [file pone.0266490.s001.docx]

August 20121

**Rebuttal letter**

**Manuscript ID: PONE-D-21-39918**

**Title: Barriers for health care access affects maternal continuum of care utilization in Ethiopia; spatial analysis and generalized estimating equation**

Tesfa Sewunet Alamneh*, Achamyeleh Birhanu Teshale, Yigizie Yeshaw, Adugnaw Zeleke Alem, Hiwotie Getaneh Ayalew, Alemneh Mekuriaw Liyew, Zemenu Tadesse Tessema, Getayeneh Antehunegn Tesema, Misganaw Gebrie Worku

**PLOS ONE**

**Dear Editor and reviewers,**

We would like to thank for your consideration and suggestion for the betterment our manuscript and make it more informative. We tried to amend the manuscript and address the questions raised by reviewer on it. Our point-by-point responses for each comment and questions are described in detail on the following pages. Further, the details of changes were shown by track changes in the supplementary document attached.

Editor’s comment

1. Please ensure that your manuscript meets PLOS ONE's style requirements, including those for file naming. The PLOS ONE style templates can be found at
   <https://journals.plos.org/plosone/s/file?id=wjVg/PLOSOne_formatting_sample_main_body.pdf> and 
   <https://journals.plos.org/plosone/s/file?id=ba62/PLOSOne_formatting_sample_title_authors_affiliations.pdf>

**Author’s response:** Thank you dear editors for your concern. We tried to amend the format of the manuscript according to the journal guidelines

2. In your Data Availability statement, you have not specified where the minimal data set underlying the results described in your manuscript can be found. PLOS defines a study's minimal data set as the underlying data used to reach the conclusions drawn in the manuscript and any additional data required to replicate the reported study findings in their entirety. All PLOS journals require that the minimal data set be made fully available. For more information about our data policy, please see <http://journals.plos.org/plosone/s/data-availability>.

**Author’s response:** Thank you dear editors for your concern. We have putted this statement on data availability part. *All the relevant data were included in the manuscript. However, it is ethically not acceptable to share the DHS data set to third parties and anyone who want the data set can access from the Measure DHS program at* [*www.dhsprogram.com*](http://www.dhsprogram.com/) *, through legal requesting. The authors had no special access privileges others would not have.*

3. We note that Figures 2 to 5 in your submission contain [map/satellite] images which may be copyrighted. All PLOS content is published under the Creative Commons Attribution License (CC BY 4.0), which means that the manuscript, images, and Supporting Information files will be freely available online, and any third party is permitted to access, download, copy, distribute, and use these materials in any way, even commercially, with proper attribution. For these reasons, we cannot publish previously copyrighted maps or satellite images created using proprietary data, such as Google software (Google Maps, Street View, and Earth). For more information, see our copyright guidelines: <http://journals.plos.org/plosone/s/licenses-and-copyright>.

**Author’s response:** Thank you dear editors for your concern. The map is not copyrighted rather we have done using GIS software based on the shape files of Ethiopia received from Ethiopian Central Statistical Agency (CSA) by explaining the purpose of the study (***https://africaopendata.org/dataset/ethiopia-shapefiles***) and GPS data (longitude and latitude) from measure DHS program by explaining the objective of the study through online requesting and allow us to access the shape file and GPS data. Now we cite the source of the shape file since it is needed to explore the spatial distribution of incomplete utilizations of maternal continuum of care.

4. Please review your reference list to ensure that it is complete and correct. If you have cited papers that have been retracted, please include the rationale for doing so in the manuscript text, or remove these references and replace them with relevant current references. Any changes to the reference list should be mentioned in the rebuttal letter that accompanies your revised manuscript. If you need to cite a retracted article, indicate the article’s retracted status in the References list and also include a citation and full reference for the retraction notice.

**Author’s response:** Thank you dear editors for your concern. We tried to change some references but we didn’t change reference from 23 up to 25 because they are books about spatial analysis.

**To reviewer 1**

1. All Figures quality may be improved (high resolution)

**Author’s response:** Thank you dear reviewer for your comment. As per your recommendation we tried to improve the quality of figures.

2. At least one additional Figure (illustration) may be provided as to highlight the summary or prospect of this study.

**Author’s response**: Thank you dear reviewer for your comment. We have illustrated it on the manuscript.

3. **Comment**: Authors should discuss about the limitation to their study.

**Author’s response**: Thank you dear reviewer, we tried to discuss the limitation of our study with in the study.

4. **Comment**:  The English of manuscript can be polished (minor)

**Author’s response:** Thank you dear reviewer for your comment. We have checked the overall manuscript regarding language usage and grammar errors as and we try to amend it. In addition, we also consult language experts in our university and amendments were done based on their comments.

**To reviewer 1**

1. Add the strength and limitations of the study in the manuscript.

**Author’s response**: Thank you dear reviewer for your comment. We have included it in the last paragraph of discussion.

2. The data is well presented in the manuscript and has undergone statistical rigor. But the author may improve the resolution of figures 1-5. The figures lack the clarity to make any conclusion. (Major)

**Author’s response**: Thank you dear reviewer for your comment. As per your comment we tried to improve the resolution of all figures.

3. The highlights the challenges and prospects of the study in the manuscript.

**Author’s response**: Thank you dear reviewer for your comment. We have tried to address it by putting the following statements; Globally, evidence on the magnitudes and determinants of maternal continuum of care has grown substantially. This information has been used as a preventive measure that is linked to maternal and child health problems. From a policy point of view, the interventions which are designed to enhance to maternal health service utilizations such as dissemination information through mass media, women empowerment by providing education, improving their wealth status and creating opportunities to have their own jobs, and tackling barriers for accessing health service is needed to enable mothers for receiving the full continuum of maternal health services.
